# Physician and patient preferences for dosing options in migraine prevention

**DOI:** 10.1186/s10194-019-0998-8

**Published:** 2019-05-09

**Authors:** Robert Cowan, Joshua M. Cohen, Erik Rosenman, Ravi Iyer

**Affiliations:** 1 Department of Neurology and Neuroscience, Stanford University School of Medicine, Stanford, Los Angeles, CA USA; 2 Teva Pharmaceuticals, Frazer, Philadelphia, PA USA; 3 Healthcare Strategy Partners LLC, Eldersburg, Baltimore, MD USA

**Keywords:** CGRP antibody, Dosing preference, Dosing flexibility, Therapy adherence, Migraine prevention, Monthly dosing, Quarterly dosing

## Abstract

**Background:**

Adherence to a therapy, though a key factor for successful treatment, is low among patients with chronic conditions such as migraine. Dose frequency plays a major role in adherence. This study evaluated the impact of having flexible dosing options on acceptance of and adherence to a new migraine preventive therapy class among adults with migraine.

**Methods:**

In this observational study, two 20-min online surveys were completed: one by physicians currently treating adult patients with migraine and the other by adults with migraine. Both surveys presented the participants with three scenarios: 1) only monthly, 2) only quarterly, and 3) both dosing options of the new medication are available. Physicians estimated the proportion of their migraine patients who would receive the new medication in each scenario. Patients were asked about their dosing preference when either or both options are available. Respondents were asked to rate the likelihood of their acceptance of and adherence to the therapy.

**Results:**

400 physicians and 417 US adults with migraine completed the surveys. The availability of both dosing options yielded a significant increase in the proportion of patients expected to receive the new medication. The overall proportion of patients favoring monthly dosing (35%) was similar to the proportion favoring quarterly dosing (40%). Among those who preferred monthly dosing (*n* = 147), a greater proportion indicated they are more likely to fill the prescription (77% vs 56%, *P* < 0.05) and remain adherent (80% vs 57%, *P* < 0.05) when only monthly is available versus when only quarterly is available. Similarly, among those who preferred quarterly dosing (*n* = 166), a greater proportion indicated they are likely to fill (63% vs 55%, *P* < 0.05) and remain adherent (62% vs 54%, *P* < 0.05) when only quarterly is available compared with when only monthly is available.

**Conclusions:**

Physicians anticipated that the proportion of patients to receive the new medication would increase when both dosing options are available. Patients stated that they are more likely to fill the prescription and adhere to the new therapy when their preferred dosing regimen is available.

## Background

Adherence to therapy (taking medication as prescribed over a period of time) is one of the key predictors of preferable health outcomes [1, 2]. However, medication nonadherence is a problem of striking magnitude that leads to poor health outcomes, higher hospitalization rates, and increased healthcare costs, especially among patients with a chronic condition [2, 3].

Adherence is influenced by a multitude of social and economic factors, such as family support, social stigma, healthcare coverage, and financial status. Other factors are disease specific (symptom severity and consistency), healthcare system specific (accessibility), treatment related (complexity, duration, and adverse events), or patient related (personality) [4]. The World Health Organization has stated that increased dosing frequency is one of the most common barriers to medication adherence [5]. Evidence from studies in a variety of disease areas, including asthma, hypertension, diabetes mellitus, depression, epilepsy, and HIV, has shown that once-daily dosing of oral medication leads to better adherence than more-frequent dosing regimens (spanning 2–4 times daily) [6, 7]. With respect to daily versus weekly dosing regimens, a study analyzing the effect of iron supplementation in pregnant women reported better adherence for weekly than for daily dosing [8]. Although these studies consistently report a beneficial effect of longer dosing intervals on medication adherence, scant data are available for more-extended intervals, such as monthly and quarterly regimens.

One disease area that will enable such a comparison to be made is migraine, a neurologic disorder characterized by attacks of severe, throbbing head pain with associated features such as photophobia, phonophobia, nausea, and vomiting [9, 10]. Recently, novel migraine preventive therapies that are administered monthly or quarterly by subcutaneous injection were approved by the US Food and Drug Administration (FDA) [11–13]. This new class of monoclonal antibodies targeting the calcitonin gene-related peptide (CGRP) pathway is efficacious and well tolerated [14–16]. Previously, migraine preventive treatment options consisted of onabotulinumtoxinA, beta-blockers, calcium channel blockers, angiotensin II receptor antagonist inhibitors, antiepileptics, and antidepressants [17, 18]; however, several studies reported low adherence to oral migraine preventive medications, with more than 80% of patients discontinuing within 1 year, mainly due to modest efficacy and side effects [19–21]. Administration of onabotulinumtoxinA in a practice setting every 3 months may allow physicians better control of patient adherence than they might have when their patients take oral medications. However, direct comparisons between daily oral delivery of preventive medication and quarterly injections of onabotulinumtoxinA have not been reported. In a population for whom adherence to preventive migraine medication has been historically low because of suboptimal efficacy and poor tolerability, it is therefore beneficial to investigate the impact of different dosing schedules on adherence when patients are offered an efficacious and well-tolerated therapy.

Choosing the best dosing schedule for patients with migraine may be challenging, as the migraine population is unique in several ways. Migraine is often accompanied by other comorbidities, such as anxiety and catastrophizing [22, 23], which may affect the extent to which patients accept and adhere to a new treatment that is delivered by a needle. In addition, for patients with chronic migraine, the presence of cutaneous allodynia and central sensitization may play a role.

Thus, to determine the optimal treatment schedule for an injectable therapy, understanding the needs of the target population is important. To our knowledge, a limited number of studies have characterized patients’ preferences for the formulation and dosing frequency of migraine preventive therapies [24, 25]. A survey of 250 American and Brazilian patients with migraine suggests that dosing frequency may be of less relevance than other factors, such as efficacy, onset of effect, and adverse events [24]. A separate survey of Greek outpatients seeking neurologic consultation for headaches found that, when asked to assume that all options had equal efficacy and safety, more migraine patients preferred a migraine preventive therapy with a once-daily oral pill option (51%) over subcutaneous or intravenous administration on a quarterly (15% and 13%, respectively) or monthly (8% and 4%, respectively) basis [25]. In this study, we evaluated the benefits of having flexible dosing options, monthly and quarterly, on physicians’ intent to prescribe and patients’ acceptance of and adherence to a new migraine preventive therapy class that is administered by subcutaneous injection.

## Methods

### Study design and participants

This observational study used two distinct 20-min anonymized online surveys to minimize the risk of bias, one completed by physicians (including headache specialists, general neurologists, pain specialists, and primary care physicians) and the other completed by patients diagnosed with migraine. Both surveys provided a product profile that contained information as follows: The medication effectively prevents migraine in adult patients with no major safety issues, is self-administered by subcutaneous injection using a prefilled syringe, and can be taken with other medication as recommended. In addition, there were three assumptions: The medication is FDA approved, its effectiveness has been confirmed in the past year while it has been on the market, and costs for the treatment are to be disregarded.

The physician survey collected information on demographics, primary specialty, specialized training, and clinical setting, as well as on the practice of treating patients with migraine, including patient volume and characteristics, and experience with current migraine treatment. The survey contained questions about the physicians’ dosing preferences and which factors they find important when deciding on a dosing regimen for their patients. To assess the impact of dosing options on the intent to prescribe the new migraine preventive therapy class, which is given as a self-administered subcutaneous injection, the physicians were presented with three scenarios: 1) only monthly dosing is available, 2) only quarterly dosing is available, and 3) both monthly and quarterly dosing options are available. Physicians were asked about their likelihood of prescribing the new preventive therapy class for their migraine patients using a seven-point Likert scale with 1 being “not at all likely” and 7 being “extremely likely”.

The patient survey collected demographic and baseline disease information, including migraine frequency and current migraine management. To assess the impact of dosing options on the acceptance of and adherence to the new migraine preventive therapy class, the patients were presented with the same three scenarios as included in the physician survey. For each scenario, using the same seven-point scale as the physician survey, patients were asked about their likelihood of filling the prescription and taking the medication consistently over 1 year. In addition, patients provided their individual dosing preferences and key factors underlying their choice.

### Inclusion criteria

Headache specialists, pain specialists, general neurologists, and primary care physicians from the US were included in this study if they had a fellowship or sub-specialty training in headache medicine or pain management and/or had a primary specialty in neurology, general/family practice, internal medicine, or anesthesiology. Additional selection criteria included that at least 50% of their patients with migraine were adults and that they consulted with at least 150 adult patients in a typical month.

Patients were included in this study if they were at least 18 years of age, were diagnosed with migraine before 2017, had at least one headache day per month in the past 6 months, and had a history of acute or preventive prescription medicine use.

### Exclusion criteria

Respondents who completed the survey in substantially less time than anticipated (physicians in < 10 min, patients in < 5 min), did not provide proper answers, or did not complete open-ended questions were excluded from the analysis. Completed surveys were checked for duplicate Internet Protocol (IP) addresses to ensure that no respondent had taken the survey twice.

### Data analysis

Data analysis included descriptive statistical analysis and comparison of means through analysis of covariance (ANCOVA) testing, with significance set at *P* < 0.05. The quantitative survey data was not weighted because the purpose of the research was to test the statistical change and impact of flexible dosing, not to estimate actual change in potential prescribing of a new class of therapy.

## Results

### Study population

A total of 400 US physicians, of whom 55 were headache specialists, 180 general neurologists and pain specialists, and 165 primary care physicians, filled out the physician survey and met the selection criteria (Table 1). On average, physicians were 49 years of age with 16.5 years in practice, and most of them were office-based (87%). More than half of their patients were private/commercially insured (53%). The proportion of patients with chronic migraine (CM) was 20%, while 23% had high-frequency episodic migraine (EM, 10–14 headache days/month), 30% moderate-frequency EM (5–9 headache days/month) and 27% low-frequency EM (1–4 headache days/month).

**Table 1:** Demographics and baseline characteristics of survey participants (physicians)

	Physicians (*N* = 400)
Age, years, mean (range)	49 (23–74)
Women, %	75
Ethnicity, %	
White/Caucasian	67
Asian/Pacific Islander	24
Hispanic/Latino	3
Black/African American	3
Years in practice, year	16.5
Patient volume, number of patients per month	
Adult patients	379
Adult patient with migraine	84
Patient type, %	
CM (≥15 headache days/month)	20
High-frequency EM (10–14 headache days/month)	23
Moderate-frequency EM (5–9 headache days/month)	30
Patient insurance, %	
Private/commercial	53
Medicare Part D	24
Medicaid	8
ACA	8
VA/TRICARE	4
Practice setting, %	
Office-based	87
Hospital-based	13

*ACA*, Affordable Care Act; *CM*, chronic migraine; *EM*, episodic migraine; *VA* Veterans Affairs

A total of 417 patients with migraine completed the patient survey and met the selection criteria for inclusion in this study (Table 2). Their mean age was 45 years, ranging from 18 to 86 years, and 78% were women. More than half of the patients had at least a college degree (54%), were employed full-time (57%) and had private/commercial healthcare insurance (82%). The proportion of participants with CM was 21%, while 25% had high-frequency EM and 55% had moderate-frequency EM.

**Table 2:** Demographics and baseline characteristics of survey participants (patients with migraine)

	Migraine Patients (*N* = 417)
Age, years, mean (range)	45 (18–86)
Women, %	78
Ethnicity, %	
White/Caucasian	88
Hispanic/Latino	8
Black/African American	5
Education level, %	
At least college	54
High school	9
Employment status, %	
Employed full-time	57
Homemaker	13
Retired	11
Annual household income before taxes ≥$75,000 in 2017, %	43
Insurance, %	
Private/commercial	82
Medicare Part D	11
ACA	4
VA/TRICARE	3
Residence, %	
Suburban	54
Rural	23
Urban/city	23
Migraine frequency, n (%)	
CM (≥15 headache days/month)	86 (21)
High-frequency EM (10–14 headache days/month)	103 (25)
Moderate-frequency EM (5–9 headache days/month)	228 (55)

*ACA*, Affordable Care Act; *CM*, chronic migraine; *EM*, episodic migraine; *VA*, Veterans Affairs

### Reasons for choosing monthly versus quarterly dosing and likelihood of physician prescribing a new class of biologics

**Fig. 1:**
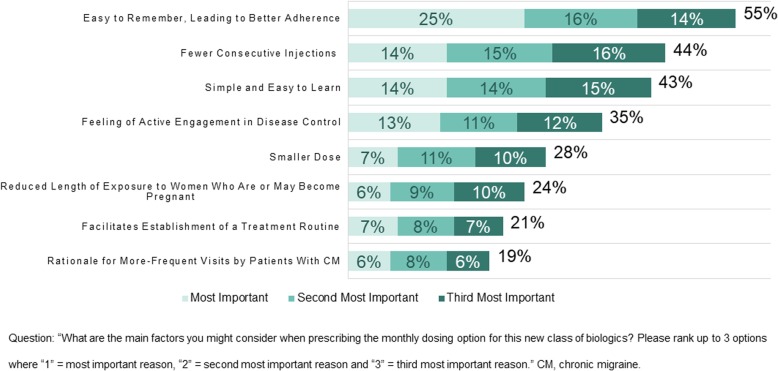
Reasons physicians choose to prescribe monthly dosing

Physicians were asked to rank their reasons for prescribing the monthly dosing option using 1 as “most important”, 2 as “second most important”, and 3 as “third most important”. Key reasons for monthly dosing (Fig. 1) included ease of remembering that may lead to better adherence (cited by 55% of physicians) and learning for patients (43% of physicians), as well as the availability of monthly dosing for those who wish to have fewer consecutive injections (44% of physicians).

**Fig. 2:**
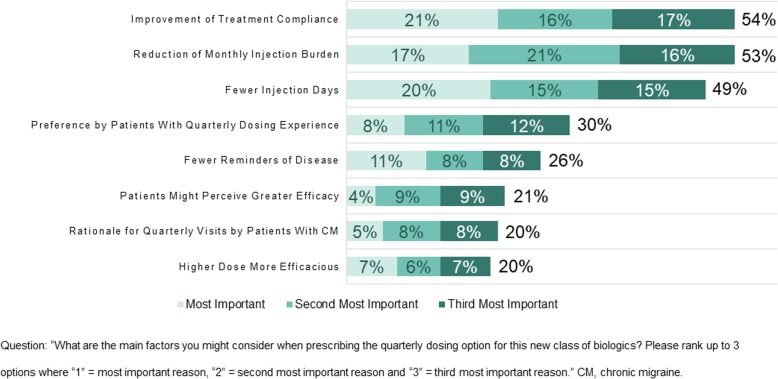
Reasons physicians choose to prescribe quarterly dosing

Using the same ranking method described for the value of monthly dosing, physicians were also asked to select the most important reasons for prescribing the quarterly dosing option. More than half of the physicians found improvement of treatment adherence (54% of physicians) and reduction of monthly injection burden (53% of physicians) to be the most important reasons for the quarterly dosing option (Fig. 2), followed by fewer injection days (49% of physicians).

**Fig. 3:**
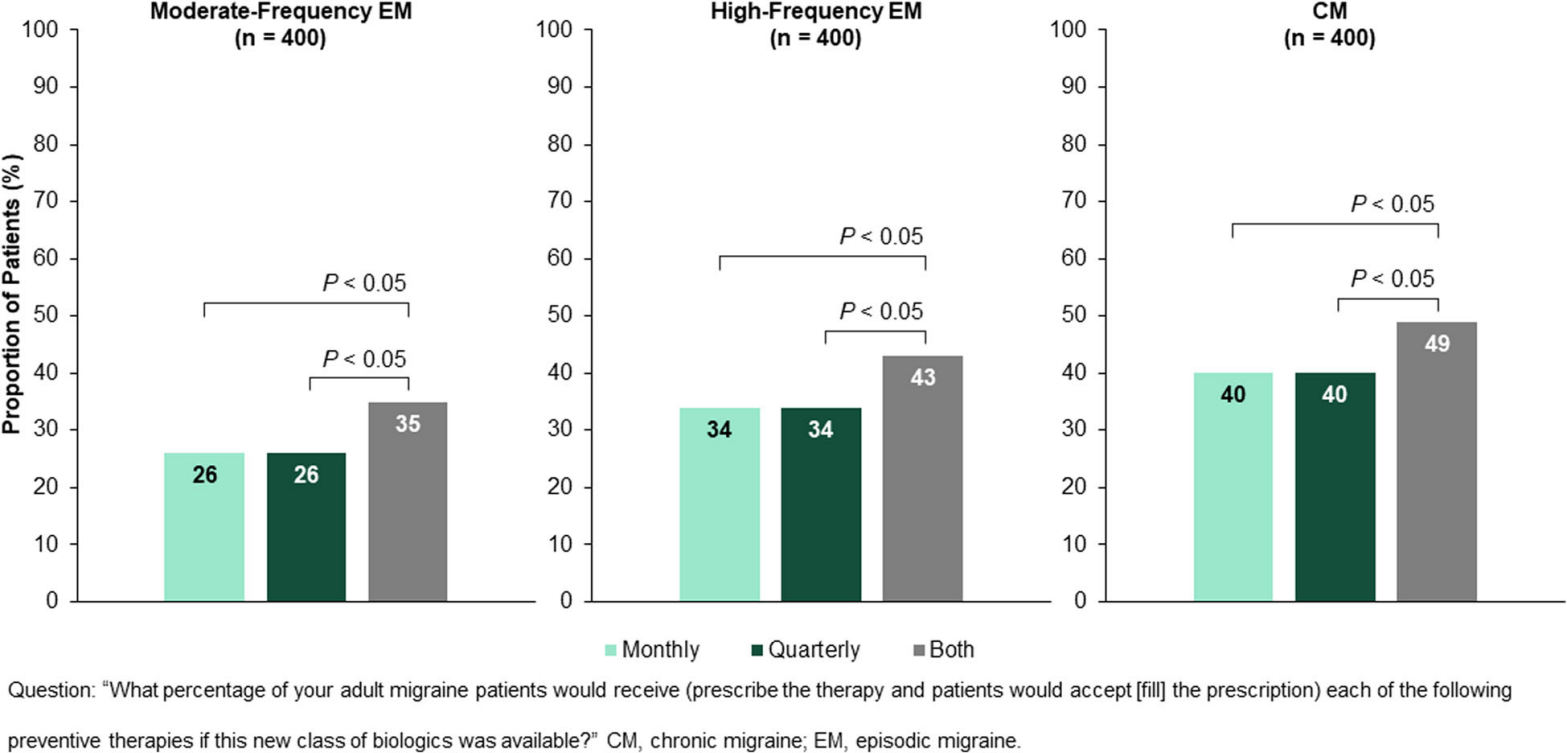
Proportion of patients expected by physicians to receive the new medication when only monthly, only quarterly or both dosing options are available

To assess the impact of flexible dosing on the number of patients receiving (physicians prescribing and patients accepting) the new medication, physicians were asked to provide their expectations in scenarios in which monthly, quarterly, or both dosing options are available (flexible dosing). When flexible dosing is available, the proportion of patients expected to receive the new medication increased significantly (all *P* < 0.05) as follows: moderate-frequency EM, from 26% to 35%; high-frequency EM, from 34% to 43%; CM, from 40% to 49% (Fig. 3). Physicians anticipated a relatively even split of prescribing across the two dosing options (mean monthly, 23.4%; mean quarterly, 26.0%) when both monthly and quarterly dosing are available for patients with CM. To decide which dosing regimen to prescribe, 95% of the physicians said that they need to know whether female patients are pregnant or plan to become pregnant, and 96% found it important to know the individual’s dosing preference and comfort level with self-injection.

### The impact of flexible dosing options on the likelihood of patient acceptance of and adherence to the new class of biologics

**Fig. 4:**
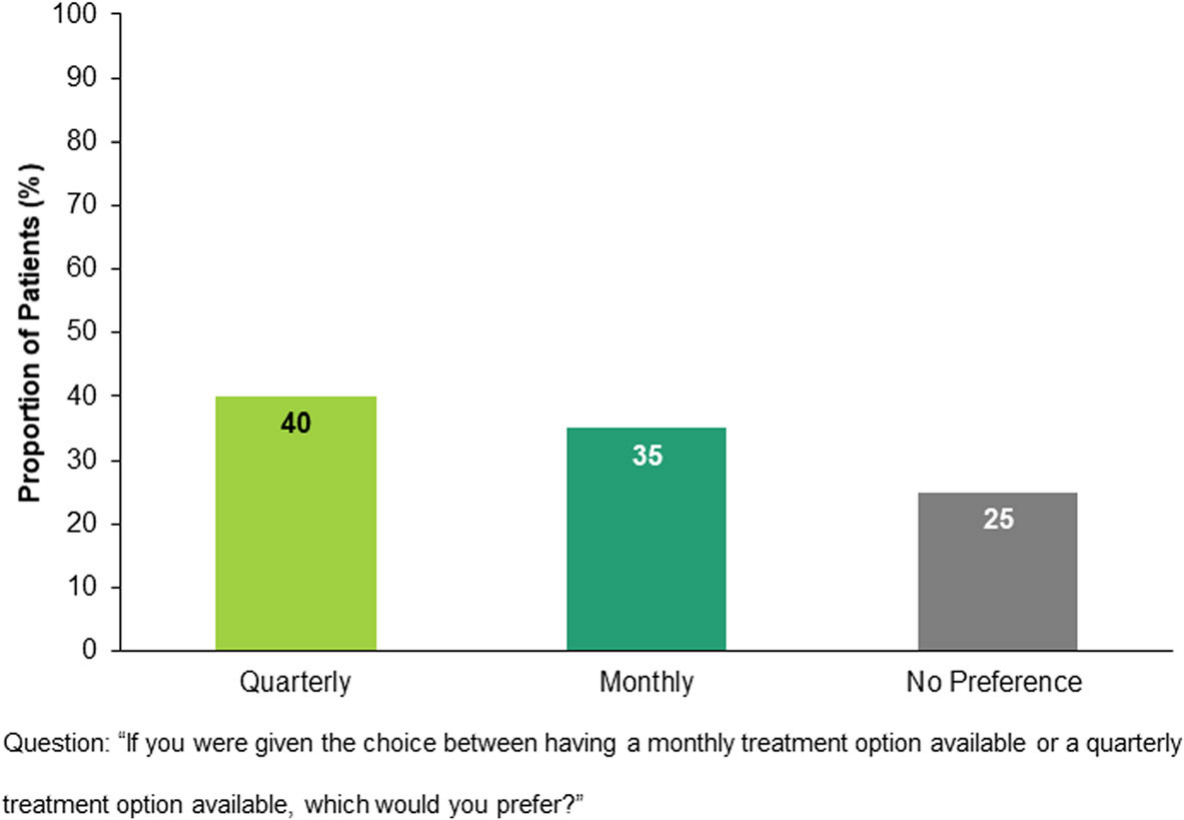
Patients preference: Monthly dosing, quarterly dosing, or on preference

Patients with migraine were asked to choose their favored dosing option in a scenario in which both monthly and quarterly dosing regimens are available for the same medication. Comparable proportions of patients preferred either monthly or quarterly dosing (35% and 40%, respectively), while 25% of patients had no preference (Fig. 4).

**Fig. 5:**
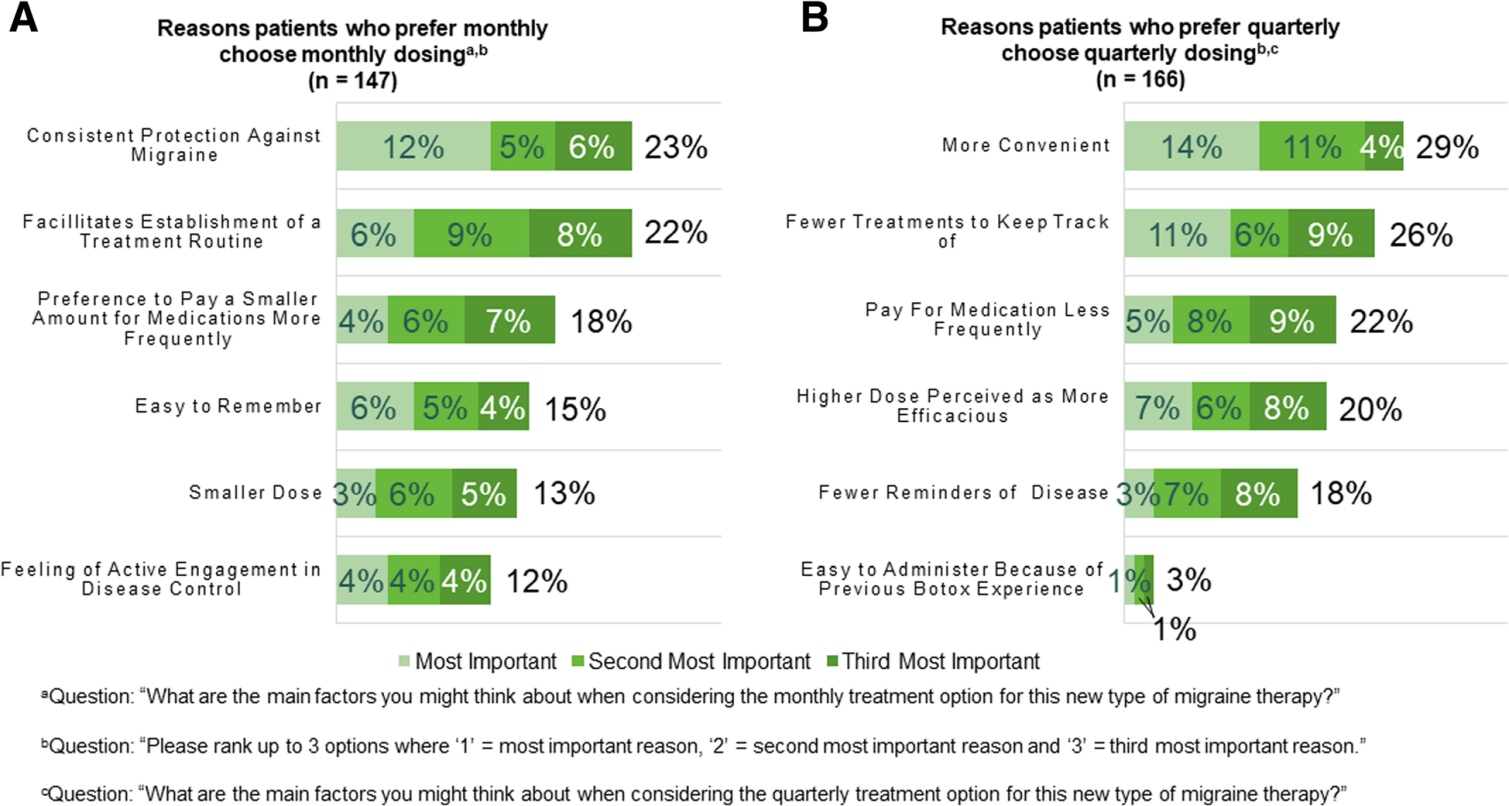
Main reasons patients prefer monthly or quarterly dosing

Similarly to physicians, patients chose the most important factors for their preferred dosing option. Patients who preferred monthly dosing found consistent protection against migraine (23% of patients) and easier establishment of a treatment routine (22% of patients) most important for the monthly dosing option (Fig. 5a). Patients favoring the quarterly dosing option chose higher convenience (29% of patients) and fewer treatments to keep track of (26% of patients) as the most important reasons for their preference (Fig. 5b).

**Fig. 6:**
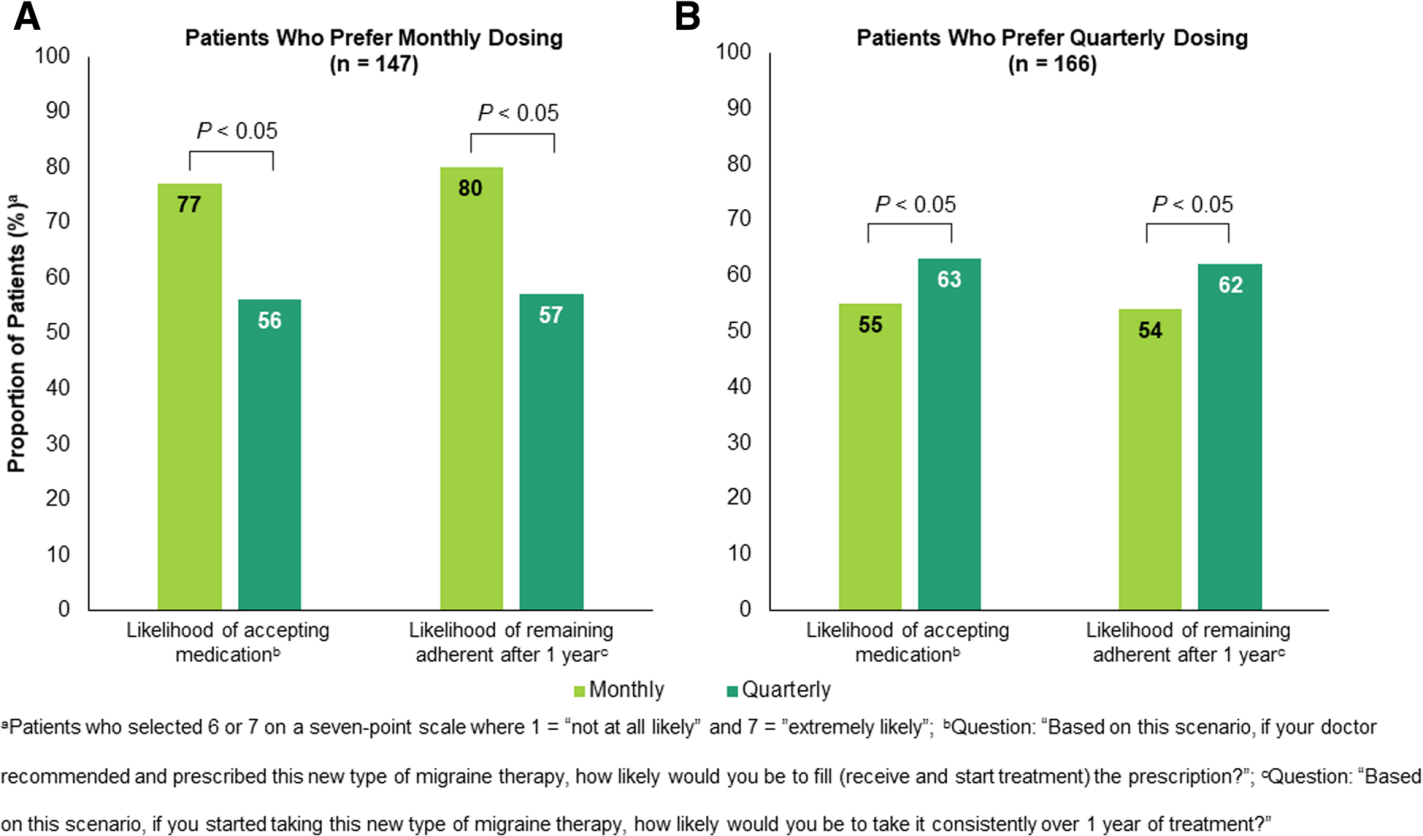
Likelihood of acceptance of and adherence to the new medication based on dosing preference and availability

Subsequently, participants ranked on a seven-point scale, with 1 being “not at all likely” and 7 being “extremely likely,” their likelihood of acceptance of and adherence to the new medication in scenarios in which either monthly or quarterly dosing is available. Among patients who preferred monthly dosing (*n* = 147), a greater proportion indicated that they would be likely to fill the prescription (77% monthly vs 56% quarterly, *P* < 0.05) and remain adherent (80% monthly vs 57% quarterly, *P* < 0.05) if only monthly dosing is available and prescribed, as compared with if only quarterly dosing is available (Fig. 6a). Likewise, a greater proportion of patients who preferred quarterly dosing (*n* = 166) stated that they would be likely to fill the prescription (55% monthly vs 63% quarterly, *P* < 0.05) and remain adherent (54% monthly vs 62% quarterly, *P* < 0.05) when only quarterly dosing is available and prescribed, as compared with when only monthly dosing is available (Fig. 6b). Among all patients, 80% responded that having both dosing options available for the same medication is important, and 25% stated that they prefer having the flexibility to choose when starting a new type of treatment as the main reason.

## Discussion

Several studies conducted on patients with chronic diseases suggest that longer dosing intervals improve adherence [6–8]. However, these studies focused primarily on daily dosing regimens. When considering monthly and quarterly dosing options for a preventive migraine therapy that is administered by subcutaneous injection, it is tempting to believe that, given the current literature, the quarterly dosing interval would lead to better adherence. However, there is a lack of evidence on long-interval dosing preferences among patients and physicians, and potential advantages of having flexible dosing options for a medication have not been explored. Thus, the ideal long-interval dosing schedule is not known.

This observational study involved two 20-min anonymized online surveys, which assessed patients’ and physicians’ attitudes toward monthly and quarterly dosing options for a new preventive migraine therapy. In addition, these surveys evaluated whether a drug with variable dosing regimens would be advantageous over a drug with only one dosing regimen, and whether there was a perceived advantage to monthly or quarterly dosing.

When asked about the value of monthly dosing, physicians indicated that this option would be simple and easy to remember for the patients. In previous studies, 42% to 58% of patients who were using monthly injectables for multiple sclerosis indicated that simply forgetting to administer the medication was the major reason for not adhering to the treatment plan [26–28]. It will be interesting to examine whether quarterly administration will have an impact on adherence over monthly dosing that is similar to what has been reported for daily versus twice- or thrice-daily dosing. Patients value monthly dosing because they would feel continuously protected against migraine and it would facilitate the establishment of a treatment routine. Thus, some patients might experience improved adherence with a monthly regimen that allows them to consistently take the new medication on a more-frequent fixed schedule.

When asked about the value of quarterly dosing, physicians indicated that this option would improve treatment adherence and would be beneficial for those who want to decrease their monthly injection burden. Similarly, some patients liked quarterly dosing because it is convenient and requires keeping track of only four injection days per year. Responses from both physicians and patients suggest that a quarterly dosing plan may be appealing to patients who have established a treatment routine, are confident with self-injection, and want the convenience of less-frequent injection days. Taken together, each dosing option, monthly and quarterly, has unique benefits that are valuable for patients depending on their individual needs and preferences.

Surprisingly, physicians had no personal dosing preference, and they found both monthly and quarterly dosing options advantageous. Physicians also indicated that it was important for them to know their patients’ lifestyles to help in determining which dosing regimen to recommend. These results indicate that physicians would be willing to prescribe the dosing option that would most likely reflect their patient’s wishes and lifestyle, rather than prescribing according to their own first preference.

Responses from the migraine population showed that similar proportions of patients preferred each dosing option. Thus, patients could benefit from having both dosing regimens available. Dosing flexibility was important for most patients, because it allows them to choose the treatment that matches their individual lifestyle. The ability to adjust the care plan without having to switch to a different medication may thus be highly beneficial for patients who experience changes in their living situation or who want to match the dosing schedule of medications for concurrent conditions.

Furthermore, when flexible dosing options are offered, the likelihood of accepting a new therapy among patients who otherwise would have not considered using this medication may increase. For example, monthly dosing may be more appealing than less-frequent quarterly dosing to patients who would like more guidance by a physician in establishing a treatment routine. Because the medication is delivered via needle [11–13], patients may recognize each injection as a de novo experience, particularly those who experience central sensitization, such as cutaneous allodynia, with their migraine [29]. Patients with anxiety or catastrophization might also prefer more-frequent intake of medication over quarterly administration to feel more protected from migraine [22, 23]. Conversely, patients who are comfortable with self-injection may want to start with quarterly injections.

Another important finding of this study is the increased likelihood of patients to accept and adhere to the medication when given their preferred dosing regimen. Patients who preferred quarterly dosing cited reasons related to convenience, while those who preferred monthly dosing cited reasons of consistency. These findings emphasize that it is important for clinicians to discuss goals of care and dosing preference with patients so that patients can participate in the choice of quarterly versus monthly dosing. This may impart a sense of control over the treatment choice that could help drive adherence.

According to the surveys, when both monthly and quarterly dosing are available, physician prescribing and patient acceptance of the anti-CGRP therapy class would be significantly higher than if only monthly or only quarterly dosing were available for patients across all types of migraine. Furthermore, the number of patients who would receive the medication increases by escalating severity of migraine, suggesting that offering both dosing options would be even more appealing for those with more-severe disease. Whether these findings can be extrapolated to other chronic conditions with similar long-term dosing options remains to be seen.

These results suggest that offering multiple dosing options for a medication may increase the number of patients receiving preventive migraine treatment and may improve therapy adherence.

One shortcoming of the study is that it did not evaluate differences among delivery systems (prefilled syringe versus autoinjector, office-administered versus self-administered, and intravenous versus subcutaneous or intramuscular). The study also did not investigate the option of switching between the two dosing regimens. For example, physicians and patients could decide to start with monthly dosing to assess efficacy or tolerability and then switch to quarterly dosing once a treatment routine has been established. In a recent survey, experts rated a similar strategy most suitable for establishing a new therapy of long-acting injectable antipsychotics in patients with schizophrenia or schizoaffective disorder. They preferred starting with a short-interval treatment (4–14 days) using an oral antipsychotic medication before transitioning to a long-acting injectable version of the same molecule with an injection interval of 4–12 weeks [30]. Future studies could investigate the impact on physician and patient preference and acceptance of having the ability to switch between dosing regimens, as well as the impact of other variables known to affect adherence.

## Conclusions

The availability of both monthly and quarterly dosing options for a therapy would benefit patients with any migraine severity. Dosing flexibility would allow patients to choose their preferred therapy based on their individual needs, with a commensurate increase in the likelihood of patients receiving and being adherent to the new migraine preventive therapy class.

## Abbreviations

ACA: Affordable Care Act
ANCOVA: Analysis of covariance
CGRP: Calcitonin gene-related peptide
CM: Chronic migraine
EM: Episodic migraine
FDA: Food and Drug Administration
IP: Internet protocol
VA: Veterans Affairs
